# Epicutaneously applied Der p 2 induces a strong T_H_2-biased antibody response in C57BL/6 mice, independent of functional TLR4

**DOI:** 10.1111/all.12399

**Published:** 2014-04-16

**Authors:** C Stremnitzer, K Manzano-Szalai, P Starkl, A Willensdorfer, S Schrom, J Singer, U Reichart, S Akira, E Jensen-Jarolim

**Affiliations:** 1 Comparative Immunology and Oncology, Department of Pathophysiology and Allergy Research, Center of Pathophysiology, Infectiology and Immunology, Medical University of ViennaVienna, Austria; 2 Comparative Medicine, Messerli Research Institute of the University of Veterinary Medicine Vienna, Medical University of Vienna and University ViennaVienna, Austria; 3 Institute of Animal Breeding and Genetics and Biomodels Austria, University of Veterinary Medicine ViennaVienna, Austria; 4 Department of Host Defense, Research Institute for Microbial Diseases, Osaka UniversityOsaka, Japan

**Keywords:** allergic dermatitis, Der p 2, house dust mite, LPS, TLR4

## Abstract

**Background:**

The major house dust mite allergen Der p 2 is a structural and functional homologue of MD-2 within the TLR4–CD14–MD-2 complex. An asthma mouse model in TLR4-deficient mice recently suggested that the allergic immune response against Der p 2 is solely dependent on TLR4 signaling. We investigated whether similar mechanisms are important for Der p 2 sensitization via the skin.

**Methods:**

In an epicutaneous sensitization model, the response to recombinant Der p 2 in combination with or without lipopolysaccharide (LPS) was compared between C57BL/6 WT and TLR4-deficient mice. We further analyzed possible adjuvant function of exogenous cysteine proteases.

**Results:**

Sensitization with rDer p 2 induced similar levels of allergen-specific IgG1 and IgE antibodies in both mouse strains. LPS increased the systemic (antibody levels, cytokine release by restimulated splenocytes) and local (infiltration of immune cells into the skin) Th2 immune responses, which against our expectations were stronger in the absence of functional TLR4 expression. Barrier disruption by papain, a protease with structural homology to Der p 1, did not enhance the sensitization capacity of rDer p 2. However, the presence of LPS increased the stability of rDer p 2 against the protease.

**Conclusion:**

Our data suggest that rDer p 2 alone can cause a strong T_H_2-biased response via the skin being enhanced in the presence of LPS. This response is not reliant on functional TLR4, but *vice versa* TLR4 expression rather protects against epicutaneous sensitization to house dust mite allergen Der p 2.

Extracts from house dust mite (HDM) *Dermatophagoides pteronyssinus* (Der p) comprise at least 23 known allergens – most of them revealing specific molecular features associated with T_H_2 skewing 1–4.

The major HDM allergen Der p 2 shows strong molecular and functional homology to MD-2, the lipopolysaccharide (LPS)-binding component of the MD-2–CD14–TLR4 complex 5,6. Der p 2 may even reconstitute TLR4 signaling in the absence of MD-2 in airway epithelia 6–12, whereas Der p 2 stimulates airway smooth muscles through TLR2 13. TLR4-deficient mice are generally unresponsive to LPS 14 and are unresponsive to inhaled Der p 2 or to airway sensitization with Der p 2 9, suggesting that the homology of Der p 2 to MD-2 is causative in the sensitization against this allergen. Therefore, TLR4 was a major factor in HDM extract-induced lung inflammation in a mouse model of asthma 10,15. Co-encounter of Der p 2 or other allergens with proteolytic allergens (cysteine or serine proteases) may support sensitization – particularly the adjuvant function of the cysteine protease Der p 1 in the respiratory sensitization to other allergens (HDM allergens as well as other allergens) has been outlined in several studies 1,16–20.

The skin is a potent and important physiologic route of sensitization to diverse allergens 21, whereas mucosal sites are rather regarded as tolerogenic 22,23. Most models of epicutaneous sensitization use the model allergen ovalbumin 24,25, intradermal or subcutaneous allergen application 26–28. We established a dermatitis model based on percutaneous application of rDer p 1 and rDer p 2 in BALB/c mice 29, where the enzymatic activity of Der p 1 was an important cofactor for sensitization via the skin. Based on this model, we investigated here the epicutaneous sensitization potential of Der p 2, specifically in the context of interaction with TLR4 and LPS, as well as the possible adjuvant function of a co-applied enzymatic allergen.

## Methods

Details for standard methods (ELISA, IHC) in Supplementary Part.

### Animals

Female TLR4^−/−^ mice (S. Akira 14) were obtained from Biomodels Austria (University of Veterinary Medicine Vienna, Himberg, Austria), originally generated on a sv129/C57BL/6 mixed genetic background, and they were further backcrossed into C57BL/6 mice for more than 8 generations 30,31. Matching female wild-type C57BL/6 mice were purchased from Charles River, Germany. Experiments were conducted according to the European Community rules for animal care with the permission number BMWF-66.009/0170-II/10b/2009 of the Austrian Ministry of Science.

### Experimental epicutaneous sensitization model

Eight-week-old mice**,** eight animals per group, were used, and the experiment was repeated twice.

For epicutaneous sensitization, backs of mice were depilated using Veet creme sûpreme (Reckitt Benckiser, Switzerland AG), devoid of any enzymes. After skin recovery for 2 days, 75 μl allergen/control solutions (Fig. 1) were applied onto filter disks (11 mm in diameter) placed in 12-mm chambers of single-chamber Finn Chamber strips. Tapes were placed onto the backs of mice for 24 h.

In total, mice were sensitized four times in 3-week intervals, and blood samples were collected prior to the 1st sensitization and 14 days after each sensitization (Fig. 1).

Three weeks after the last treatment, mice were challenged using the same doses of allergens or controls, but applied with Q-tip instead of Finn Chamber strips. 6 h after the challenge, mice were killed and final blood was collected by heart puncture. Skin biopsies were taken for histochemistry and spleens isolated for splenocyte preparation.

### Degradation assay (SDS-PAGE and silver staining)

To follow degradation of rDer p 2 by papain, the following concentrations were used: Der p 2: 1 mg/ml; Papain: 0.3 mg/ml (when combined with rDer p 2) or 1 mg/ml (alone or with inhibitor); LPS: 2.5 μg/ml; 25× molar E-64 inhibitor and incubated alone or in combination as indicated in Fig. 6C. 18 μl of each sample was incubated with 7 μl 4× denaturating SDS-PAGE sample buffer for 5 min at 95°C. 15 μl of sample was separated by 15% SDS-PAGE and silver-stained.

### Statistical analysis

All statistical tests were performed using one-way anova, comparing the mean of each group with the mean of each other group and using Tukey test as post hoc test. Data analysis was carried out, and graphs were plotted with GraphPad Prism 6 software (GraphPad, San Diego, CA, USA). In graphs, mean with SEM is shown. Multiplicity-adjusted *P*-values <0.05 were considered statistically significant. * indicates *P* < 0.05; **P < 0.01; ***P < 0.001, otherwise no statistical significance was reached.

## Results

### Epicutaneously applied rDer p 2 triggers high Der p 2-specific IgE and IgG1 antibody levels and LPS acts as cofactor in a TLR4-independent manner

Wild-type C57BL/6 and TLR4^−/−^ mice (background C57BL/6) were epicutaneously sensitized with 100 μg endotoxin-reduced rDer p 2 (Fig. S1) 29 (cp Methods S1) with or without LPS (Fig. 1). Der p 2-specific IgG1 and IgE were detectable 2 weeks after the last sensitization in both WT and TLR4^−/−^ mice. Although not all mice reacted consistently to the endotoxin-reduced rDer p 2, it was *per se* able to specifically induce T_H_2-type antibodies (Fig. 2A).

**Figure 1:**
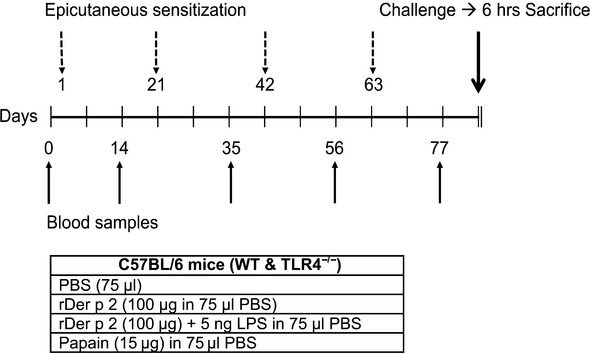
Adjuvant-free epicutaneous sensitization model for major house dust mite allergen Der p 2 and treatment groups. Wild-type C57BL/6 and TLR4^−/−^ (background C57BL/6) mice were sensitized by application of allergens onto the backs using Finn Chambers, four times in 3-week intervals, followed by a final challenge using a Q-tip. Concentrations of used allergens per animal for each sensitization round and for the challenge is indicated in the box. Each group consisted of eight animals, two independent experiments were performed.

**Figure 2:**
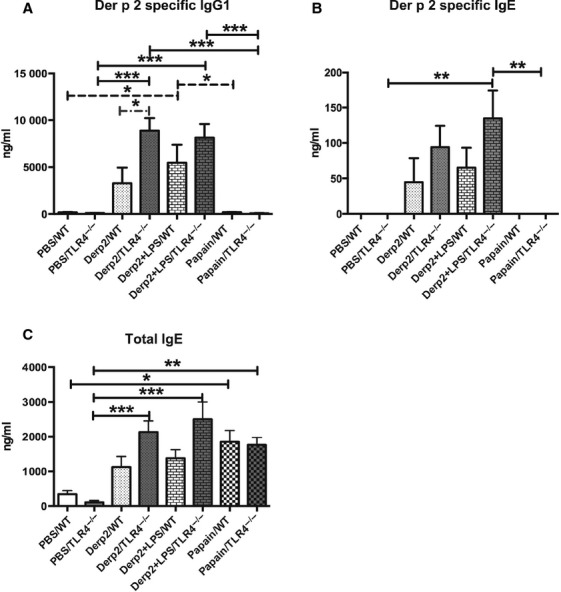
Antibody levels specific to rDer p 2 and total IgE in sera of sensitized mice indicate a T_H_2-biased immune response. rDer p 2-specific antibody levels: Serum of all animals was collected before sensitization and after each sensitization. After the last immunization, levels of rDer p 2-specific IgG1 (A) and IgE (B) antibodies as well as total IgE (C) were determined by ELISA by testing all single mice in duplicates. Mean ± SEM; *P < 0.05; **P < 0.01; ***P < 0.001.

Lacking an important component of the T_H_1 signaling axis, TLR4-deficient mice exhibited even higher levels of specific T_H_2 antibodies. IgG1 antibody titers were significantly increased compared with PBS and allergen (papain) control and WT group (Fig. 2A); total IgE (Fig. 2B) was significantly increased compared with controls. No IgG2a and – although increased – no significantly altered levels of IgG2b and IgG3 between treatment groups could be detected (Fig. S2) and were low compared with IgG1 titers. In papain-treated mice (allergen control), no Der p 2-specific antibodies were detectable.

The addition of LPS to rDer p 2 resulted in enhanced T_H_2-biased antibody responses in WT (specific IgG1, IgE, and total IgE: Fig. 2A–C) and TLR4-deficient mice (specific IgE and total IgE; Fig. 2B–C), thus independent of TLR4.

### rDer p 2 triggers a T_H_2-biased cytokine release by spleen cells *in vivo*

In spleen cells from rDer p 2-challenged TLR4-deficient and wild-type mice, release of IL-2, IL-5, IL-13, and TNF-α upon restimulation with rDer p 2 (Fig. 3), concanavalin A (positive control), and medium only (negative control) was assessed (Fig. S3). Control groups (PBS, papain) did not produce any of these cytokines or only at background levels (TNF-α) upon rDer p 2 stimulation. WT mice showed increased levels of IL-2 upon rDer p 2 stimulation, but compared with PBS controls, significantly more IL-2 was measured in supernatants from TLR4-deficient mice.

**Figure 3:**
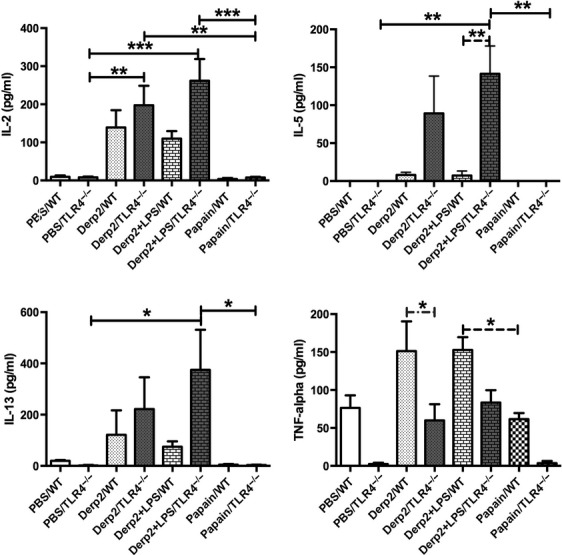
Isolated restimulated splenocytes from sensitized mice produce notable amounts of cytokines IL-2, IL-5, IL-13, and TNF-α. Splenocytes were isolated after challenge and stimulated *ex vivo* with plain culture medium containing rDer p 2. To measure cytokine release, splenocyte supernatant was collected after 72 h of cultivation and stimulation. Levels of IL-2, IL-5, IL-13, and TNF-α after restimulation with rDer p 2 were determined by ELISA (mean ± SEM; *P < 0.05; **P < 0.01; ***P < 0.001). Further *ex vivo* stimulations were carried out with plain culture medium (negative control; Fig. S3B) or plain culture medium containing concanavalin A (Con A, positive control, Fig. S3A), rDer p 2 + LPS or papain (data not shown).

Compared with PBS controls, T_H_2 cytokines IL-5 and IL-13 were significantly released from stimulated splenocytes of TLR4-deficient mice sensitized with rDer p 2 + LPS, but only in low amounts from rDer p 2-only-treated TLR4^−/−^ mice (Fig. 3). In WT splenocytes, only low levels of IL-5 and IL-13 were detectable in both rDer p 2-treated groups without an effect by LPS addition.

TNF-α release was stronger by WT splenocytes than from TLR4^−/−^ splenocytes.

### Topical rDer p 2 induces alterations in the structure of dermis and epidermis

Cutaneous hyperplasia, hyperkeratinization, and leukocyte infiltrate are characteristics of chronic cutaneous T_H_2 inflammation 32,33. Evaluation of skin samples showed an increase in epidermal layers in all rDer p 2-treated groups independent of addition of LPS or TLR4 expression (Fig. 4A). Increase in dermal thickness after allergen treatment was faint, but measurable.

**Figure 4:**
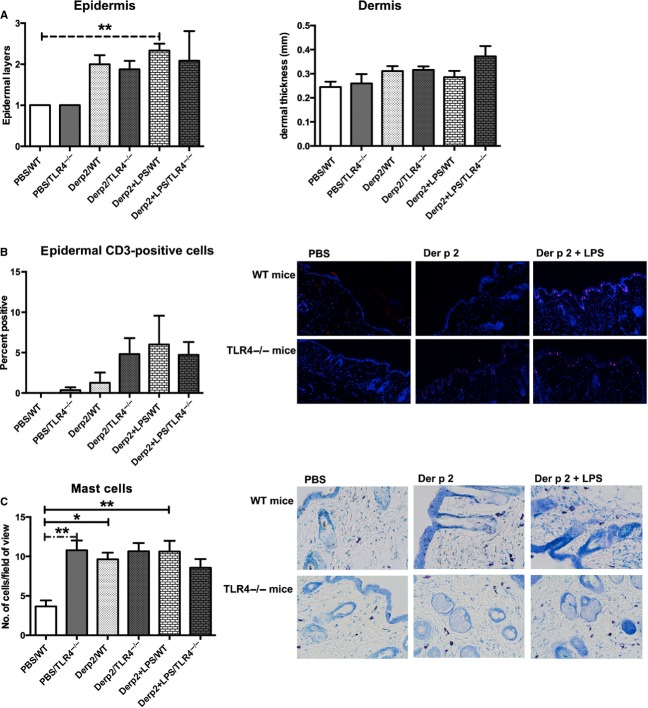
Epicutaneous sensitization and challenge altered the structure of rechallenged skin and increased mast cells and CD-3^+^ cell numbers. Paraffin-embedded, H&E-stained skin samples of sensitized and rechallenged mice (*n* = 7–8/group) were evaluated for number of epidermal layers and dermal thickening, in a blinded manner (epidermal layers) and using HistoQuest analysis program (dermis). (B) Infiltration of CD3^+^ T cells (*n* = 5/group) in the epidermis (mean ± SEM; *P < 0.05; **P < 0.01; ***P < 0.001) was evaluated by immunofluorescence (IF), on the right: representative data from one field from the skin of one mouse per group; ×20. Calculated in percent positive (CD3 + -positive) of all cells. (C) Infiltration of mast cells (*n* = 7–8/group) in the dermis (mean ± SEM; *P < 0.05; **P < 0.01; ***P < 0.001), calculated by no. of cells per field of view; on the right: representative data from one field from the skin of one mouse per group; ×40 (Giemsa staining).

Compared with PBS controls, allergen treatment and addition of LPS increased CD3^+^ cell numbers in both mouse strains. In TLR4^−/−^ mice, both rDer p 2-treated groups showed an equal increase in CD3^+^ cell numbers (Fig. 4B).

In WT mice, allergen treatment increased mast cell numbers in the dermis without further effects upon addition of LPS (Fig. 4C). In TLR4^−/−^ mice, mast cell numbers were already significantly increased in the PBS-treated group (compared with WT controls) and allergen treatments did not further increase those numbers. An increase in eosinophils could not be observed (data not shown).

### Langerin+ cells infiltrate the dermis of WT, but not TLR4-/- mice, and number of Ly6G+ cells increases with cofactor LPS

DCs, especially Langerhans cells in the epidermis and dermal dendritic cells in the dermis, are important APCs and shape immune responses 34. In WT as well as in TLR4^−/−^ mice, no differences in Lang^+^ cell numbers in the epidermis were observed, but in WT mice, rDer p 2 as well as rDer p 2 + LPS increased numbers of dDCs in the dermis by trend (Fig. 5).

**Figure 5:**
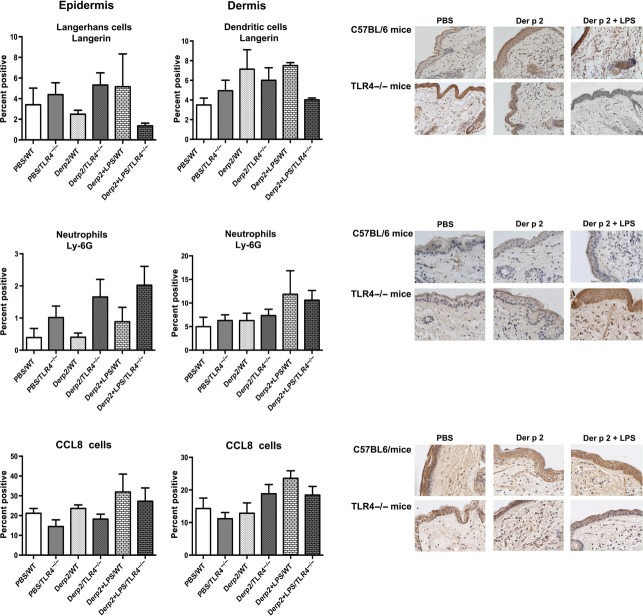
rDer p 2-sensitized mice exhibit an increase in CCL8-positive cells and invasion of dendritic cells and neutrophils in dermis and epidermis after rechallenge. Paraffin-embedded skin sections (*n* = 4–5/group) were stained with anti-Langerin, anti-Ly 6G (to detect neutrophils), and anti-CCL8 antibodies, scanned and evaluated using HistoQuest analysis program. Epidermis and dermis were evaluated separately (mean ± SEM). IHC stainings for CCL8 (×63), Ly6G (×63), and Langerin (×40): Representative data from one field from the skin of one mouse per group.

In TLR4-deficient mice, epicutaneous application of rDer p 2 increased percentage of Lang^+^ cells, while rDer p 2 + LPS reduced Lang^+^ cells.

Treatment with rDer p 2 alone did not alter dermal Ly-6G-positive neutrophil numbers of both mouse strains but increased neutrophils in the epidermis of TLR4^−/−^ mice (Fig. 5). LPS addition to the allergen rDer p 2 increased the neutrophil percentage in the dermis and the epidermis of both mouse strains.

### Mouse CCL8 is increased after topical application of rDer p 2

The CCR8–CCL8 (MCP-2) axis plays a major role in the chronic inflammatory process in response to allergens 32,35,36 by specifically acting on highly differentiated T_H_2 cells and recruiting them into skin and draining lymph nodes. Skin sections revealed no significant differences between the treatment groups, although a trend toward increased CCL8-positive cells after challenge with allergen + LPS was observed. In WT mice, dermis and epidermis comprised higher levels of CCL8-positive cells compared with TLR4-/- mice (Fig. 5). CCL8-positive cells were increased in both strains in the epidermis after restimulation with rDer p 2 + LPS. In TLR4-deficient mice, rDer p 2 alone led to more infiltration of CCL8-positive cells in the dermis and was not further altered by LPS.

### Enzymatically active papain does not boost the immune response to rDer p 2

Exogenous proteases such as Der p 1, a cysteine protease being structurally and functionally homologous to papain, have been shown to facilitate the uptake of allergens by cleaving tight junction proteins of lung epithelium, thereby enhancing specific allergic responses to concomitantly encountered allergens 12,19,20,37,38.

To test this possible adjuvant function in the skin, WT mice were pretreated with active or inactivated, E-64-pretreated papain, a structural and functional homologue to Der p 1, and applied 30 min later rDer p 2 (Table S1A).

Mice pretreated with active papain showed no increase in rDer p 2-specific IgE and IgG1 antibody titers compared with mice treated with rDer p 2 alone and less than in mice treated with rDer p 2 + LPS (Fig. 6A), and inhibition of papain’s enzymatic activity did even slightly increase the specific antibody response compared with the group pretreated with active papain, indicating that exogenous proteases were not a major factor in epicutaneous sensitization to rDer p 2.

**Figure 6:**
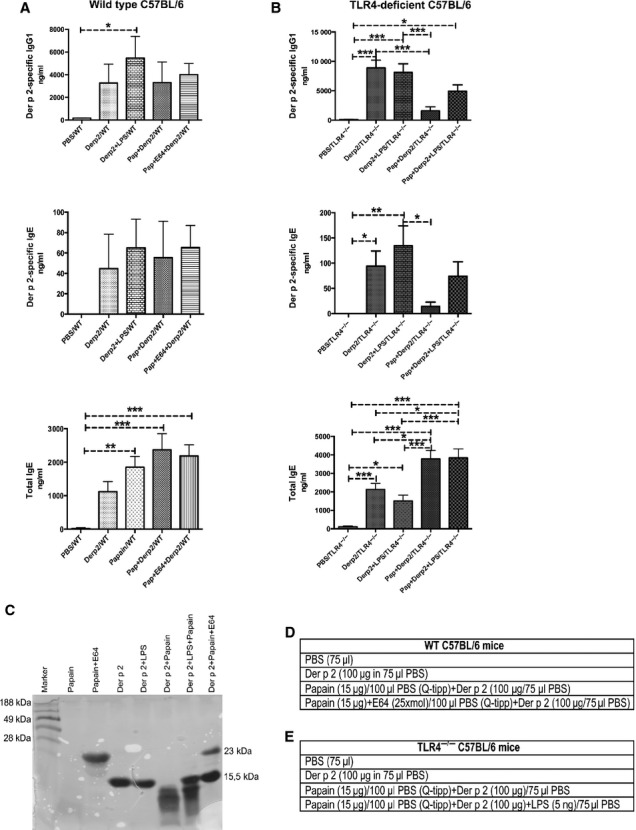
The enzymatically active papain does not show any adjuvant function for rDer p 2 in epicutaneous sensitization. (A) WT C57BL/6 mice were sensitized with enzymatically active cysteine protease papain or inactive papain and after 30 min co-sensitized with rDer p 2. After four sensitization rounds, antibody levels were measured by ELISA: Serum of all animals was collected, and rDer p 2-specific IgG1 and IgE were tested for each mouse in duplicates. To evaluate the full immune response, other IgG subclasses were tested (data not shown), but only induced in very low background levels (mean ± SEM; *P < 0.05; **P < 0.01; ***P < 0.001). (B) TLR4^−/−^ mice were sensitized with papain using a Q-tip and subsequently co-sensitized with rDer p 2 or rDer p 2 + LPS. After four sensitization rounds, antibody levels in serum collected from individual mice were measured by ELISA: rDer p 2-specific as well as papain-specific (not shown) IgG1 and IgE were tested for each mouse in duplicates (mean ± SEM; *P < 0.05; **P < 0.01; ***P < 0.001). Other IgG subclasses were tested as controls (data not shown). (C) Degradation of rDer p 2 by papain and inhibitory role of LPS. rDer p 2 or rDer p 2 + LPS was incubated with activated or E-64 (a cysteine protease-specific inhibitor)-inactivated papain. After several time points, aliquots were taken and loaded onto SDS-PAGE. Silver staining was performed to follow degradation of rDer p 2 by papain. Shown here: time point 3 h. (D) Table: Treatment groups of epicutaneous sensitization model to test the adjuvant function of the cysteine protease papain. Concentrations of used allergens per animal for each sensitization round and for the challenge are listed. Each group consisted of eight animals. Two independent experiments were performed. (E) Table: Sensitization groups to test the adjuvant function of papain and the adjuvant function of the cofactor LPS in TLR4-deficient mice (vgl. D).

### LPS as cofactor decelerates enzymatic degradation of rDer p 2

In a separate set of experiments, we evaluated the TLR4-independent effect of LPS as a cofactor in the protease (papain)-pretreated mice (Table S1B).

As observed in WT mice (Fig. 6A), rDer p 2-specific antibody titers were not increased after papain pretreatment. Specific IgG1 was even significantly decreased compared with the rDer p 2-only group, and LPS addition to rDer p 2 before application increased rDer p 2-specific IgG1, whereas total IgE levels were not influenced by LPS addition (Fig. 6B). By trend, the same result was observable in rDer p 2-specific IgE.

To test whether LPS might increase rDer p 2 stability, we incubated endotoxin-free rDer p 2 and rDer p 2 + LPS with papain for 3 h at 37°C and separated aliquots at several time points (Fig. 6C). Immediately after co-incubation (data not shown), we could detect the digestion of rDer p 2 by papain. Degradation was reduced if LPS was added to rDer p 2, at all time points 0, 1, 2, and 3 h (Fig. 6C; only time point 3 h shown). In controls, no degradation of rDer p 2 could be observed.

## Discussion

Lipopolysaccharide endotoxin is found in high concentrations in all nonsterile environmental setting and can act as adjuvant in allergic sensitization. More than 50% of all known major allergens are lipid-binding proteins 39. Several studies have shown a crucial role of TLR4 in sensitization to aeroallergens 1,7,10,13,27,40 and especially in sensitization to rDer p 2 9 via the bronchoepithelial route.

Although humans are at least 6 h per day directly exposed to HDM allergens via the skin 41, the molecular mechanism of Der p 2 in percutaneous sensitization has not been addressed yet. In contrast to data generated for the lung, our data strongly suggest that in the skin, TLR4 does not account for sensitization to rDer p 2. In our model (Fig. 1), TLR4-/- mice showed an even stronger overall T_H_2-biased antibody response to epicutaneous rDer p 2 than WT mice receiving the same treatment (Fig. 2). In contrast to lung models 7,9, LPS increased the overall immune reaction to allergen sensitization and allergen challenge via the skin apparently independent of TLR4 signaling. Sensitization to rDer p 2 was even more pronounced in TLR4-/- mice if LPS was present, and we therefore even suggest a protective role for functional TLR4, which should be addressed in future experiments and with other lipid- (and endotoxin-)binding allergens. An alternative target for further investigations could be TLR2 13 or inflammasome activation. Inflammasome proteins such as NLRP3 are abundant in the skin 42, and recent studies imply activation of the inflammasome by LPS independent of TLR4 43,44. Those alternative pathways involved in LPS recognition might overcompensate for the lack of functional TLR4, thereby triggering a stronger response in TLR4-deficient mice. The lack of TLR4, an innate immune receptor usually more prone to increase T_H_1 response, might also explain the strong bias toward T_H_2 antibody formation. LPS addition also resulted in increased numbers of CD3^+^ T cells, neutrophils (Fig. 4), and APCs in the epidermis of WT and TLR4^−/−^ mice and although being not significantly altered, implicating immune cell activation and activation of immune receptors other than TLR4, or simply an increased stability of rDer p 2 when complexed with LPS. Whereas active protease papain induced a significant decrease in specific antibodies against Der p 2 alone in TLR4^−/−^ mice, complexing Der p 2 to LPS ‘rescued’ this response.

*In vitro*, a remarkable prolongation of degradation was observed when LPS was added to rDer p 2 (Fig. 6C). Likely, other lipid-binding allergens might acquire increased stability when bound to endotoxins and should be subject of further investigation.

In our epicutaneous model, T lymphocytes were not only recruited to the skin, but also participated in the specific systemic immune response. In sensitized TLR4-deficient mice, restimulation of splenocytes with rDer p 2 significantly induced IL-2, indicating a high activation level of T cells 45 compensating the impairment of the LPS–TLR4–TNFα axis 46. In line, we observed high levels of IL-13 and IL-5 release in these mice, which are probably driven by high IL-2 levels and STAT5 activation 47,48.

We observed an overall higher baseline number of mast cells, neutrophils, CD3^+^ and – except for the Der p 2 + LPS group – lang^+^ cells in skin biopsies of TLR4-deficient mice, and marginally less CCL8^+^ cells (Figs5). The observed decrease in lang^+^ cells in both epidermis and dermis could indicate already a stage of enhanced activation and migration to the skin-draining lymph nodes.

Our results also show that the adjuvant function of exogenous proteases on the entry of other allergens may be more complex in epicutaneous sensitization than in lung models 19. Co-application of the experimental model protease papain to rDer p 2 on the skin did not enhance the immune reactivity of rDer p 2, and specific inhibition of enzymatic activity by E-64 resulted even in slightly higher rDer p 2-specific antibody titers (Fig. 6A). This might be due to simultaneous inhibition of endogenous cysteine proteases by E-64, resulting in protection of Der p 2 from degradation by endogenous proteases prolonging the encounter of intact allergen to skin-resident immune cells.

Interestingly, atopic individuals are known for genetic polymorphisms in endogenous proteases (e.g., SPINK5) and altered skin pH 49,50, relevant for the activity of proteases. The importance of genetic polymorphisms is strongly supported by our own earlier findings when comparing immune responses of BALB/c vs. C57BL/6 mice after percutaneous sensitization with rDer p 1, rDer p2 and after papain-pretreatment (K. Szalai, C. Stremnitzer, T. Kopp, A. Lukschal, I. Pali-Schoell, E. Jensen-Jarolim, unpublished data).

Whereas in BALB/c mice, typical phenotypic changes (increase in epidermal layers, dermal thickening, increase in eosinophils, but no increase in mast cells) could be observed upon percutaneous rDer p 1 and/or rDer p 2 sensitization 29, there were no significant changes observable in this regard in C57BL/6 mice (Fig. S4B) – except an increase in mast cells (not shown).

Moreover, the response toward rDer p 1, rDer p 2, and papain was different. Whereas in BALB/c mice, pretreatment with the cysteine proteases rDer p 1 or papain was crucial for the sensitization potential to rDer p 2 (Fig. S4A; 29), this was reverse in C57BL/6 mice (Fig. S4A). This finding is in accordance with the results of the present study (Fig. 6A) and with data from an i.p. immunization mouse model 51 and indicates that the genetic background of different mouse strains might alter and influence the response toward house dust mite antigens. Further studies might elucidate corresponding polymorphisms with possible implications for humans. Another interesting finding was that C57BL/6 mice have significantly higher TEWL (transepidermal water loss) levels than BALB/c mice, indicating a less effective skin barrier (Fig. S4C).

From our data, we conclude that the skin as an important sensitization route may differentially modulate a consecutive immune response than the bronchial epithelium. Other immune receptors than TLR4 are responsible for triggering epicutaneous allergic sensitization, and a knockdown of TLR4 does not necessarily protect from allergic sensitization. On the contrary, we are prompted to state that functional TLR4 might have a protective function against skin allergens.

## Supporting Information

Methods S1 Expression and purification of recombinant Der p 2.

Figure S1 Circular dichroic analysis and IgE-binding capacity of recombinant Der p 2.

Figure S2 IgG subclasses specific to rDer p 2.

Figure S3 Isolated restimulated splenocytes from sensitized mice produce cytokines IL-2, IL-5, IL-13, and TNF-α.

Figure S4 BALB/c and C57BL/6 mice react differently toward percutaneous sensitization of rDer p 1 and rDer p 2.

Table S1A Treatment groups of epicutaneous sensitization model to test the adjuvant function of the cysteine-protease papain.

Table S1B Sensitization groups to test the adjuvants function of papain and the adjuvant function of the cofactor LPS in TLR4-deficient mice. Concentrations of used allergens per animal for each sensitization round and for challenge are listed. Each group consisted of 8 animals. Two independent experiments were performed.
