# Supplementary material for: Epicutaneously applied Der p 2 induces a strong TH2-biased antibody response in C57BL/6 mice, independent of functional TLR4
Source: Allergy. 2014 Apr 16;69(6):741–51. doi: 10.1111/all.12399 (PMC4023119; doi:10.1111/all.12399)
Supplement: Supplementary file 1 [file all-69-741-sd1.docx]

**Supplementary Material**

**ONLINE METHODS (SUPPL. METHODS)**

**Expression and purification of recombinant Der p 2**

Recombinant his-tagged Der p 2 (rDer p 2) was generated using pPICZα vector, transfected into the X-33 yeast strain *Pichia pastoris* (Invitrogen; EasySelect^TM^ Pichia Expression Kit; cat.no.K1740-01). BMGY (Buffered Glycerol-complex Medium) medium was used for preculture conditions and BMMY (Buffered Methanol-complex Medium) for stimulation (Invitrogen User Manual). Stimulation was continued for 3-5 days by adding 0.5% methanol every 24 hours. After rDer p 2 purification using Ni-NTA agarose beads (Quiagen; cat.no. 30210) and Poly-Prep Chromatography Columns (Bio-Rad; cat.no. 731-1550), eluates were pooled and incubated for 30 minutes with 80% ammoniumsulphate for denaturation in 10% precipitation buffer (stock: 0,1 M sodiumphosphate buffer, pH 7,4) at 4°C. Precipitated rDer p 2 was centrifuged, pooled and transferred into dialysis membrane (Thermo Scientific; cat.no. 88243) and dialysed against distilled water at 4°C. Refolded rDer p 2 was endotoxin-purified (Hyglos, EndotrapRed, cat.no. 321063) and lyophilized. LPS content/activity was evaluated by THP1-XBlue cells (Invivogen; cat.no.thpx-sp).

Concentration of rDer p 2 was determined by BCA Protein Assay (Thermo Scientific, cat.no. 23225) and by SDS-PAGE (12%), using BSA as a control protein.

**Analysis of secondary structure by Circular Dichroism**

Circular dichroism (CD) spectroscopy was performed as previously described (27).

Briefly, measurements of our generated rDer p 2 as well as rDer p 2 from an independent laboratory (for control purposes; kindly provided from Prof. JM Saint-Remy, Leuven, Belgium) were performed with a J-715 spectropolarimeter (Jasco, Gross-Umstadt, Germany) at 0.1 mg/ml in H_2_O using a 1 mm path length quartz cuvette (Hellma, Müllheim, Germany) equilibrated at 20°C. Spectra were measured at a scan speed of 50 nm/min and 0.2 nm resolution. The average of three scans was taken as result after subtraction of the baseline spectrum. The results were calculated as mean residue ellipticity (θ) at the respective wavelengths, analyzed using dichroweb program and compared to references from literature (Tanyaratsrisakul et al., 2010).

**Testing rDer p 2-specific antibody levels by enzyme-linked immunosorbent assay (ELISA) and western blot**

To test the correct antigenicity of generated rDer p 2 on ELISA, microtiter plates (Maxisorp, Nunc, Roskilde, Denmark) were coated with rDer p 2 or rDer p 2-control, rAra h2 and ovalbumin as negative controls, in a concentration of 0.2μg per well (50mM NaHCO_3_, pH 9.6). After blocking using TBST/1% bovine serum albumin (BSA), serum of house dust mite allergic patients and non-allergic control individuals (ethic number 419/2011) were diluted 1:100 for IgG and 1:10 for IgE and incubated overnight at 4°C. For detection, directly labeled horse radish peroxidase (HRP)-labeled goat anti-human-IgE or IgG antibody (Kierkegaard & Perry, Gaithersburg, Maryland, diluted 1:3000) were used. Detection was performed using ABTS (Sigma, Vienna, Austria) at 1mg/ml in citric acid buffer (70 mM Citric acid, 125 mM Na_2_HPO_4_, pH 4.0) containing 0,03% H_2_O_2_. Optical density (OD) was determined at dual wavelengths 405/490 nm using a microplate reader (Tecan, Groedig, Austria).

To test the IgE binding capacity of newly expressed rDer p 2 on western blot, rDer p 2 was separated on 12% SDS-PAGE under denaturing conditions and transferred under semi-dry-transfer conditions onto methanol-activated PVDF membrane (Millipore, Billerca, Massachusetts). After blocking with 5% DMP (dry milk powder) in 0.5%TBST for 1 h at room temperature, incubation was continued with allergic and non-allergic patients sera, diluted 1:10. HRP labelled monoclonal mouse anti-human IgE antibody (clone HP6029, Southern Biotech, Birmingham, Alabama) diluted 1:2000 was used for detection. Bound antibody was detected using SuperSignal West Pico chemiluminescence substrate (Pierce, Rockford, Illinois).

**Monitoring endotoxin content of rDer p 2 preparations by THP1-XBlue cells**

THP1-XBlue cells (Invivogen; cat.no.thpx-sp), were cultured according to the manufacturers protocol. Cultured cells were plated with 1x10^5^ cells/well in 200 µl (RPMI-1640 medium, supplemented with 10% heat-inactivated FCS, 2mM L-Glutamine, 1% Pen/Strep and 5% Zeocin) on a 96 round-bottom well plate.

10µl/well allergen dilutions (concentrations from 1µg/well to 10 µg/well were tested) were incubated in duplicates overnight at 37°C and 5% CO_2_. As positive control different concentrations of LPS 1 µg – 5 µg – 10 µg/well (Sigma) were used, sterile PBS and medium served as negative control.

After overnight incubation 180µl/well of QUANTI-Blue (Invivogen Catalog number: rep-qb2) solution were mixed with 20 µl/well of THP1-XBlue cell supernatants into a 96 flat-bottom well plate and incubated overnight at 37°C and 5% CO_2_.

OD values were determined using a microplate reader (Tecan, Groedig, Austria) at 620-655nm.

**Monitoring specific and total antibody formation of PIS (pre-immune-serum) and 4^th^ MIS (immune-serum) by ELISA**

rDer p 2 or papain (as negative control) was coated at a concentration of 200 ng/well in a MaxiSorp 96 well flat-bottom plate (Nunc, Rochester, New York). After 2 hours blocking (1 h 37°C, 1 h 4°C) with 1% BSA/TBS/0.05% Tween20 (TBST), wells were incubated with single mouse sera, diluted 1:20 for IgE, 1:200 for IgG1, IgG2a, IgG2b, IgG3 in 0.1% BSA/TBST overnight at 4°C. For detection, primary isotype-specific rat-anti-mouse antibodies (BD Pharmingen, Schwechat, Austria) diluted 1:1000 in TBST/0.1% BSA (1 hr 37°C, 1 hr 4°C) and secondary horse radish peroxidase-labeled goat anti-rat antibody (GE Healthcare, Buckinghamshire, UK) 1:1000 (1 hr 37°C, 1 hr 4°C) were used.

Reaction was measured analogously as already described above. Antigen-specific antibody concentrations were calculated based on a mouse IgG (Southern Biotech, Birmingham, Alabama; cat.no. IgG1: 0102-01, IGg2a: 0103-01) or IgE (BD Pharmingen; cat.no. 554118) standard.

To test total IgE content in ELISA, plates were coated with 2 µg/ml anti-mouse IgE (BD Pharmingen cat.no. #553413). After blocking with 1% BSA/TBST at 4°C overnight, wells were incubated with mouse sera, diluted 1:100 in 0.1%BSA/TBST. 50 µl/well biotin anti-mouse IgE (BD Pharmingen #553419) at 2 µg/ml in dilution buffer (0,1%BSA/TBST) were used for detection and incubated for 60 minutes at room temperature. As secondary antibody, horseradish-peroxidase labeled avidin (eBioscience, cat.no. 00-4100-94) diluted 1:250 in 0.1%BSA/TBST was used and incubated at RT for 30 minutes.

For detection, TMB solution (BD Bioscience) was used as described in manufacturer’s instructions.

Additional controls were included in ELISA to test the mouse-sera against an irrelevant his-tagged allergen (his-Ara h 2; negative control; data not shown) and recombinant Der p 2 without his-tag (positive control; data not shown).

**Splenocyte stimulation and cytokine analysis**

Spleens were excised and single cell suspensions from each mouse spleen prepared immediately. Tissue was minced and filtered through a 70 µm nylon mesh (BD Biosciences, San Jose, California) and resuspended in RPMI 1640 medium (Gibco) containing glutamine and antibiotics (Pen/Strep). Erythrocytes were lysed by incubation with ACK cell lysis buffer (10mM Tris, pH 7,4; 0,83% NH_4_Cl) for 3 minutes. Lysis reaction was stopped by addition of complete growth medium, RPMI 1640 supplemented with glutamine, antibiotics and 10% fetal bovine serum (FBS). Splenocytes were counted and 4x10^6^ cells/ml cell suspension was seeded in triplicates in 96-well round bottom plates (Corning). For stimulation, 40 µg/ml rDer p 2, 40 µg/ml papain (Sigma), 5 ng/ml LPS, medium only as negative control and 5 µg/ml concanavalin A as positive control, were added. Cell supernatants were harvested after 72 h of incubation at 37°C and stored at -20°C for further analysis.

Furthermore, supernatant triplicates were pooled for each mouse. Cytokines like mouse IL-2, IL-13, and TNF-α were analyzed undiluted in duplicates, using ELISA Ready-SET-Go kits (eBioscience, Vienna, Austria) according to the manufacturer’s instructions.

**Histological analysis**

At the end of the sensitization protocol, mice were sacrificed and skin biopsies of the treatment area were taken from each mouse. Skin samples were fixed in 3.7 % neutral paraformaldehyde over night and embedded in paraffin. Paraffin-sections were cut to 5 µm thickness using a microtome (Histocom, WR. Neudorf, Austria). For haematoxylin and eosin staining, standard protocols were used as described in (Szalai et al., 2012).

For Giemsa staining, paraffin-fixed specimens were deparaffinized, rehydrated and incubated for 18 minutes in freshly prepared Giemsa solution (1:3 dilution with A. bidest.) of stock: Giemsa-Azur-Eosin-Methylenbluesolution (Merck, cat.no.: 1.09204.0500). For differentiation, object slides were washed in 0.1 – 1% acetic acid (3-6 drops of 100% acetic acid in 100ml dH_2_O). After re-dehydration in ethanol absolute and xylene, sections were embedded with entellan (Merck; cat.no. 1.07961.0500).

**Immunofluorescence**

For immunofluorescence stainings, skin sections were carefully deparaffinised (Xylol 2 x 5 min, EtOH 100% 2 x 5 min, EtOH 96% 2 x 5 min, EtOH 70% 5 min, EtOH 50% 5 min, EtOH 30% 5 min, Aqua dest. 5 min, PBS 2 x 5 min) and antigen retrieval was performed (5 minutes, 37°C)with 30 U/ml Proteinase K in PBS (Sigma, cat.no. P4850). After permeabilization (PBS/0.2%Tween20 for 5 minutes) and blocking (10% goat serum/PBS for 30 minutes at RT) anti-CD3-antibody (AbD Serotec, USA, MCS 1477), diluted 1:50 in 5 % goat serum/PBS was added and incubated over night at 4°C. On the next day, slides were washed 3 times with PBS and incubated with goat-anti-rat antibody (AF568; lifeTechnologies, cat.no.A11077) (diluted 1:1000 in PBS) and incubated for 60 minutes at RT and afterwards stained with DAPI (1:2000 in aqua dest.) for 10 minutes. After final washing steps, slides were mounted with fluoromount-G (eBioscience, cat.no. 00-4958-02) and analyzed by light microscope. Quantification was calculated after blinded evaluation.

**Immunohistochemistry**

Deparaffinization and antigen retrieval of skin sections were performed as described for immunofluorescence staining. Residual peroxidase activity was blocked by a15 minute incubation with 1% H_2_O_2_ in PBS. For CCL8 and Langerin staining, a permeabilization step (PBS/0.2%Tween20 for 5 minutes) was included. Further staining steps were performed using Santa Cruz Biotechnology, Inc. ImmunoCruz^TM^ rat ABC staining system (cat.no.sc-2019) and Santa Cruz Biotechnology, Inc. ImmunoCruz^TM^ rabbit ABC staining system (cat.no.sc-2018) according to the manufacturer’s instructions. Used 1^st^ antibodies were: rat anti-mouse Ly-6G antibody 1:100 (Novus Biological; cat.no. NBP2-00441), rabbit anti-mouse CCL8/MCP-2 antibody 1:100 (antibody-online, cat.no. ABIN676298), rabbit anti-mouse Langerin antibody 1:250 (Novus Biological, cat.no. NB100-56733) and controls: rat IgG2b (Abcam, cat.no.ab18541), rabbit IgG (abcam, cat.no. ab27478). Primary antibodies were incubated over night at 4°C for Ly-6G and CCL8 and for 2 hours at RT for Langerin-staining.

After DAB staining, slides were counterstained with filtered, undiluted haematoxylin for 5 minutes, washed with aqua dest. and PBS, dehydrated and embedded with entellan. Slides were evaluated using HistoQuest® cell analysis software from TissueGnostics, Vienna, Austria.

**Animals (Suppl.Fig.4)**

Female wild-type C57BL/6 and BALB/c mice from Charles River, Germany were used. Experiments were conducted according to the European Community rules for animal care with the permission number BMWF-66.009/0170-II/10b/2009 of the Austrian Ministry of Science. Sensitization protocols and analysis were performed as previously described (Szalai et al., 2012).

**Measurement of TEWL**

WT C57BL/6 and BALB/c-mice (n=6 mice/group) were depilated on their backs using Veet creme sûpreme (Veet® Sûprem’Essence Easy Spray, Reckitt Benckiser Switzerland AG). Two days after depilation, TEWL was measured using Tewameter®TM300 (Courage+Khazaka electronic GmbH) 3 times/mouse for 30 seconds and mean of all 3 measurements were used for analysis using T-TEST.

**SUPPLEMENTARY FIGURES**

**Suppl.Fig.1: Circular Dichrois analysis and IgE-binding capacity of recombinant Der p 2**

To verify correct folding and structure of generated and purified recombinant Derp 2, CD analysis was performed and compared to the literature (A). Recognition of rDer p 2 by serum IgE of atopic, HDM-allergic patients was tested in ELISA and Westernblot and compared to control serum from non-allergic patients.

**Suppl.Fig.2: IgG-subclasses specific to rDer p 2**

rDer p 2-specific antibody levels: Serum of all animals was collected before sensitization and after each sensitization. Levels of rDer p 2-specific IgG2a, IgG2b and IgG3 antibodies of single mice were determined by ELISA (mean ± SEM; *, P < 0.05; **, P < 0.01; ***, P < 0.001).

**Suppl.Fig.3: Isolated restimulated splenocytes from sensitized mice produce cytokines IL-2, IL-5, IL-13 and TNF-α**

Isolated splenocytes were stimulated *ex vivo* with plain culture medium (negative control) or culture medium containing Con A (positive control). Levels of IL-2, IL-5, IL-13, and TNF-α after restimulation with rDer p 2 were determined by ELISA (mean ± SEM; *, P < 0.05; **, P < 0.01; ***, P < 0.001).

**Suppl.Fig.4: BALB/c and C57BL/6 mice react differently towards percutaneous sensitization of rDer p 1 and rDer p 2**

**A:** BALB/c mice were sensitized with rDer p 2 alone or after pretreatment with rDer p 1 (Szalai et al., 2012) or 15 µg papain/mouse. rDer p 2-specific IgG1 antibody levels after last sensitization round are shown (mean+SEM).

**B:** Pilot-study testing percutaneous sensitization of rDer p 2 and rDer p 1 in C57BL/6 mice (n=5 mice/group). Pretreatment with rDer p 1 did not increase rDerp2-specific antibodies as in the same model using BALB/c mice. Contrary to BALB/c mice, eosinophil numbers were not significantly increased and increase of epidermal layers was seen to a lesser extent (compare to (Szalai et al., 2012)).

**C:** Measurement of Transepidermal water loss at basal levels comparing BALB/c and C57BL/6 mice (n=6 mice/group, 2 separate experiments). C57BL/6 mice had significantly higher TEWL values (p<0,05), indicating a less effective epidermal barrier (mean+SEM is shown).

**References**

Szalai, K., T. Kopp, A. Lukschal, C. Stremnitzer, J. Wallmann, P. Starkl, L. Vander Elst, J.M. Saint-Remy, I. Pali-Scholl, and E. Jensen-Jarolim. 2012. Establishing an allergic eczema model employing recombinant house dust mite allergens Der p 1 and Der p 2 in BALB/c mice. *Experimental dermatology* 21:842-846.

Tanyaratsrisakul, S., N. Malainual, O. Jirapongsananuruk, W.A. Smith, W.R. Thomas, and S. Piboonpocanun. 2010. Structural and IgE binding analyses of recombinant Der p 2 expressed from the hosts Escherichia coli and Pichia pastoris. *International archives of allergy and immunology* 151:190-198.
