# Supplementary material for: Epicutaneously applied Der p 2 induces a strong TH2-biased antibody response in C57BL/6 mice, independent of functional TLR4
Source: Allergy. 2014 Apr 16;69(6):741–51. doi: 10.1111/all.12399 (PMC4023119; doi:10.1111/all.12399)
Supplement: Supplementary file 2 [file all-69-741-sd6.docx]

Stremnitzer & Szalai *Suppl. Table 1*

**Suppl.Table 1A**

**WT (C57BL/6) mice**

PBS (75µl)

Der p 2 (100µg in 75µl PBS)

Papain (15µg) in 100µl PBS (Q-tipp) + Der p 2 (100µg in 75µl PBS)

Papain (15µg) + E64 (25xmol) in 100µl PBS (Q-tipp) + Der p 2 (100µg in 75µl PBS)
